# Supplementary material for: Inhibition of transcription by dactinomycin reveals a new characteristic of immunogenic cell stress
Source: EMBO Mol Med. 2020 Apr 23;12(5):e11622. doi: 10.15252/emmm.201911622 (PMC7207166; doi:10.15252/emmm.201911622)
Supplement: Supplementary file 1 — Appendix [file EMMM-12-e11622-s001.pdf]

Appendix

Inhibition of transcription by dactinomycin reveals a new characteristic of immunogenic cell stress

Juliette Humeau, Allan Sauvat, Giulia Cerrato, Wei Xie, Friedemann Loos Francesca Iannantuoni, Lucillia Bezu, Sarah Lévesque, Juliette Paillet, Jonathan Pol, Marion Leduc, Laurence Zitvogel, Hugues de Thé, Oliver Kepp, Guido Kroemer

Table of content

- Appendix Fig S1 IC_{60} of predicted ICD inducers.
- Appendix Fig S2 Validation of the eIF2α kinases knock out.
- Appendix Fig S3 Gating strategy for quantification of phagocytosis.
- Appendix Fig S4 Gating strategy for quantification of $MHCII^+$ and $CD86^+$ cells among $CD11c^+$ cells.
- Appendix Fig S5 Transcription and translation inhibitors are predicted as ICD inducers.
- Appendix Fig S6 Sequence for the eIF2α knock-in mutation.
- Appendix Table S1 Transcription and translation inhibition data for the custom anti-cancer library.
- Appendix Table S2 Transcription inhibition for the positive and negative hits selected with the artificial intelligence module.
- Appendix Table S3 ICD scores of specific categories of agents.
- Appendix Table S4 DNA sequences for U2OS eIF2αS51A and $eIF2AK^{-/-}$ generation.
- Appendix Table S5 Statistical tests and p-values.

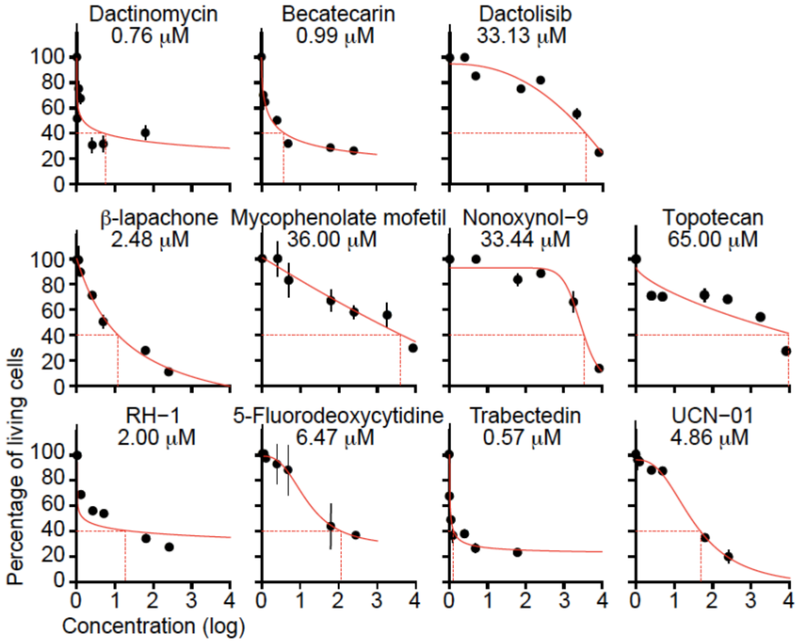

Appendix Figure S1

Appendix Figure S1. IC_{60} of predicted ICD inducers.

To determine the IC_{60} (concentration of the agent that reduces viability to 40 %), we treated human osteosarcoma U2OS wild-type cells for 24 h with a range of concentrations from 0.01 to 5 µM for dactinomycin and trabectedin; from 0.05 to 10 µM for becatecarin, RH-1, β-lapachone and 5-fluorodeoxycytidine; from 0.5 to 50 µM for dactolisib, UCN-01, topotecan and mycophenolate mofetil and from 1 to 100 µM for nonoxynol-9. Then cells were stained with Hoechst 33342 and propidium iodide (PI) and assessed by automated microscopy. The percentage of living cells (normal-sized, $Hoechst_{low}$, PI_{-}) is presented after logarithmic-transformation ($\ln(concentration_{\mu M} + 1)$) for each concentration. One representative experiment with quadruplicates is depicted as means ± SD. A log-logistic regression was performed to determine the IC_{60}. The indicated IC_{60} values are means of one to three independent experiments.

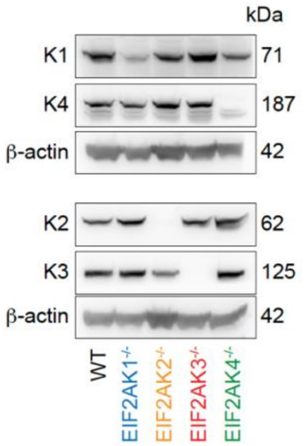

Appendix Figure S2

Appendix Figure S2. Validation of the eIF2α kinases knock out. U2OS cells were knocked out for each of the four eIF2α kinases using the CRISPR-Cas9 technology. One knock-out clone was selected for each kinase and was further validated by immunoblot with HRI (K1) and GCN2 (K4) on one and PKR (K2) and PERK (K3) on a parallel blot. Both membranes were further probed with anti β-actin antibody as a loading control.

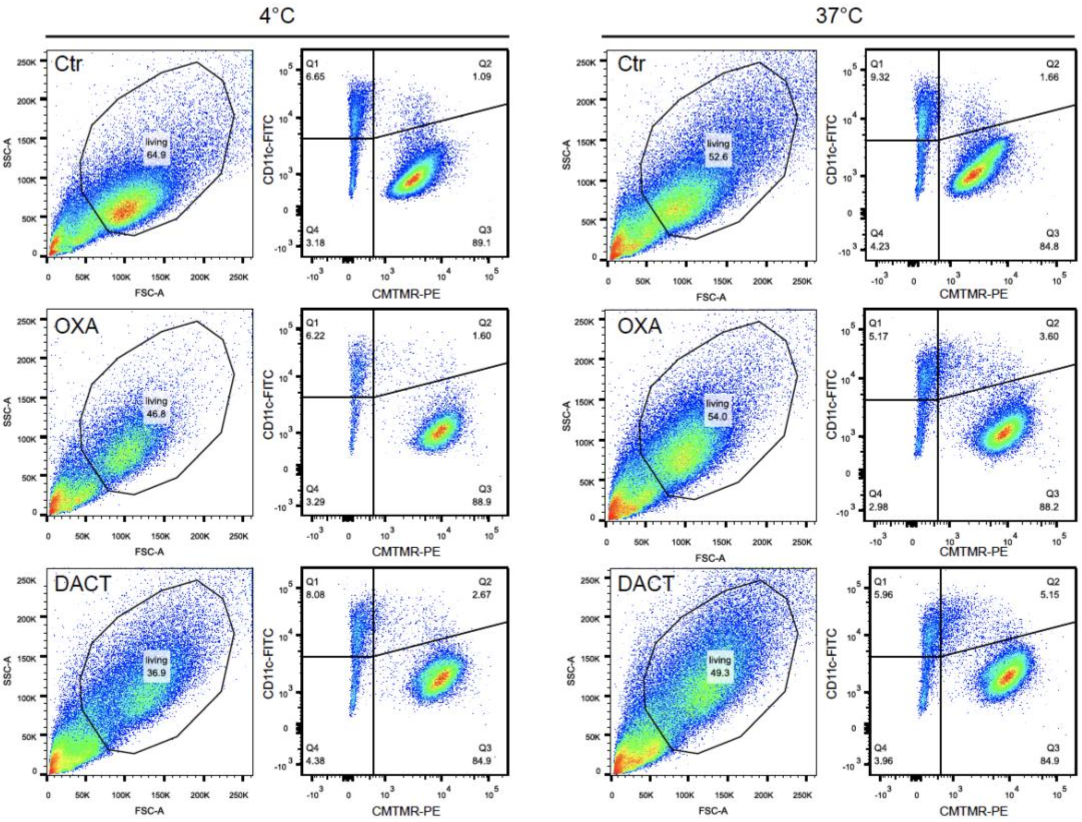

Appendix Figure S3

Appendix Figure S3. Gating strategy for quantification of phagocytosis. Mouse fibrosarcoma MCA205 cells were stained with CellTracker Orange (CMTMR) and treated for 24 h with 1 µM dactinomycin (DACT) or 500 µM oxaliplatin (OXA) as a positive control. Then, dying MCA205 were co-cultured with differentiated bone marrow derived dendritic cells (BMDCs) for 4 h at 37 °C or at 4 °C. Cells were collected and dendritic cells were stained with CD11c specific antibody before analysis by flowcytometry. After exclusion of debris, thresholds were applied to determine $CD11c^{+}$ and $CMTR^{+}$ cells, with double positive cells representing the events of phagocytosis.

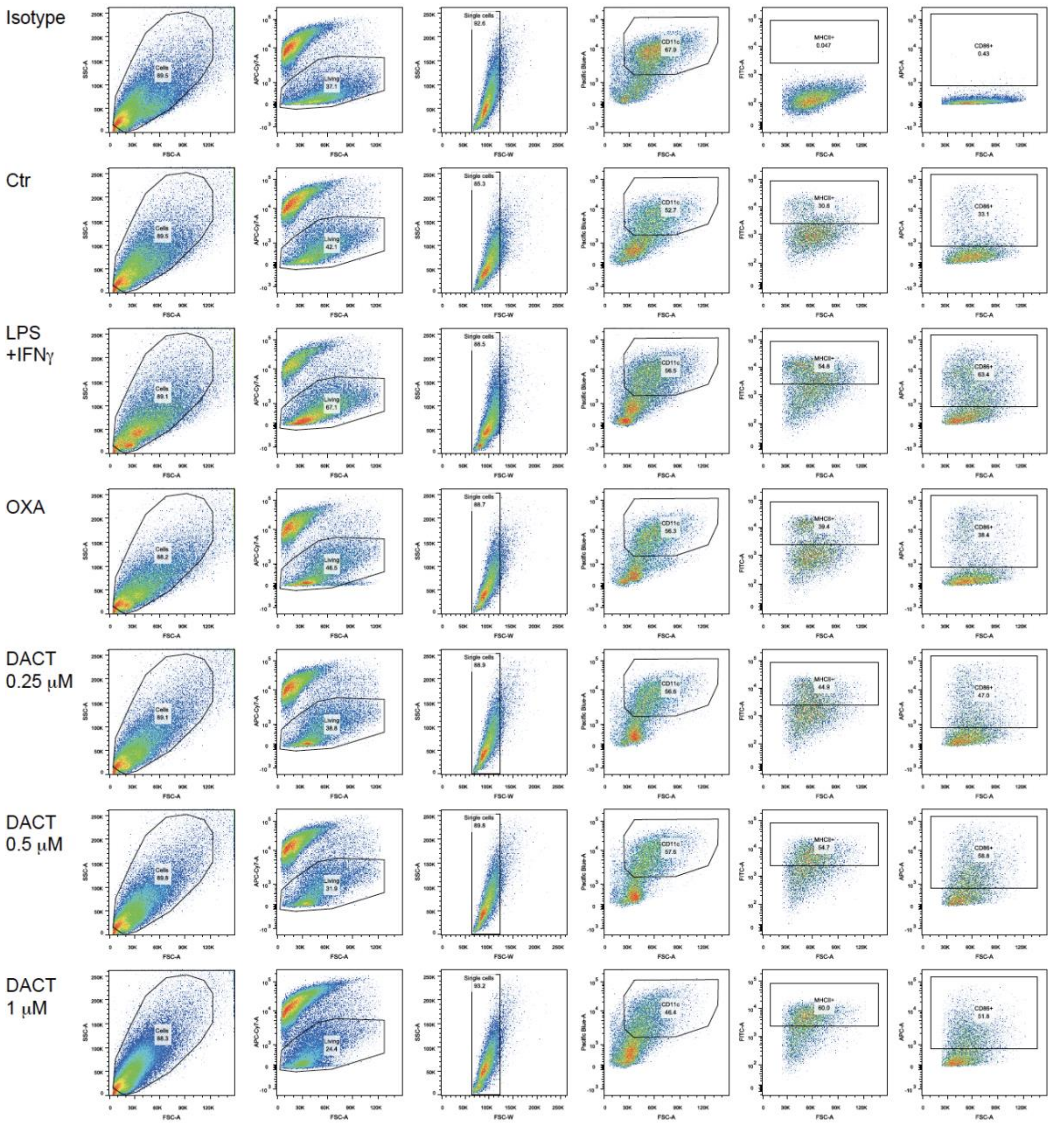

Appendix Figure S4

Appendix Figure S4. Gating strategy for quantification of MHCII$^+$ and CD86$^+$ cells among CD11c$^+$ cells. Mouse fibrosarcoma MCA205 cells were treated for 24 h with 500 µM oxaliplatin (OXA) or with 0.25, 0.5 or 1 µM dactinomycin (DACT). Then, dying MCA205 were co-cultured with differentiated bone marrow derived dendritic cells (BMDCs) for 24 h at 37 °C. As a positive control, BMDCs were co-cultured with 1 µg/mL LPS and 100 ng/mL IFNγ. Cells were collected and stained with LIVE/DEAD marker, as well as with CD11c, MHCII and

CD86 specific conjugated antibodies before analysis by flowcytometry. After debris exclusion, living single cells were selected based on the LIVE/DEAD staining and SSC-A/FSC-W distribution. Finally, the percentages of MHCII+ and CD86+ among CD11c+ cells were determined.

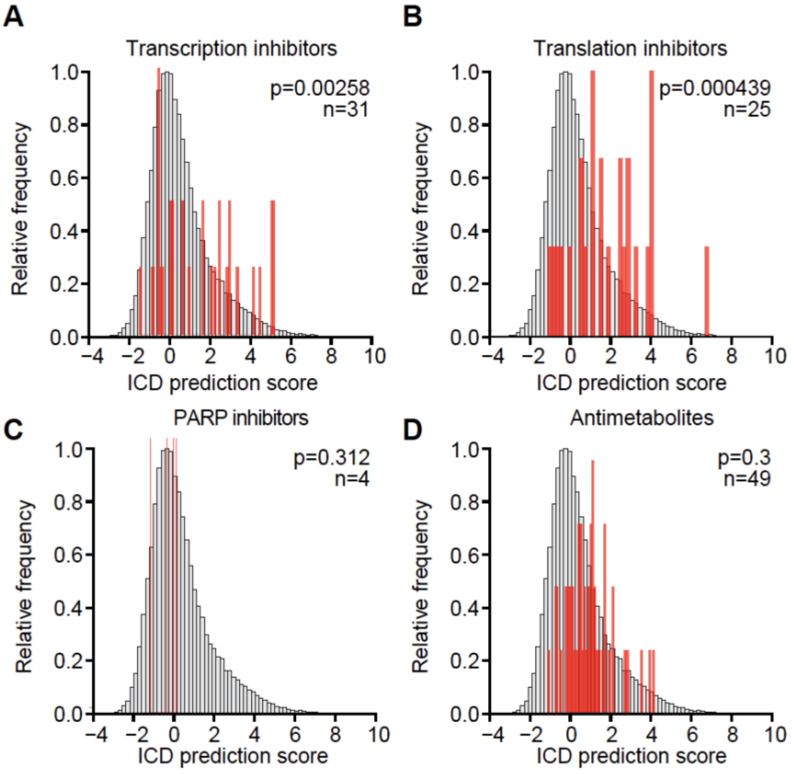

Appendix Figure S5

Appendix Figure S5. Transcription and translation inhibitors are predicted as ICD inducers. The 50,000 compounds of the NCI-60 library were annotated for different parameters including transcription and translation inhibition. The predicted ICD score was calculated with a previously described model built by artificial intelligence (Bezu et al, 2018). Data as in **Fig. 8 I-L** are depicted here as frequency normalized to the maximum frequency in relation to ICD scores. The distribution for all compounds is plotted in grey. In red, compounds falling into categories of interest are depicted which are transcription inhibitors (n=31) (**A**), translation inhibitors (n=25) (**B**) as well as two other random categories chosen as controls, PARP

inhibitors (n=4) and antimetabolites (n=49) (**C, D**). The p-values (p) calculated with Kolmogorov-Smirnov test are indicated on each graph.

eIF2αS51A:

```
   1  TTTTTCCTTT ATATAGTGAG TGGTAGCGTA TACAATGTTT GCTCACTTCG
  51  GCAAAGAGTA GCTTTAGTTT TCTGATTAGT TATAATAGTG ATGCTTCCCA
 101  TCATTTGATA TGCCTTAAAG TAGTATTTTA CTCTTGAGGT ATTCCTTTAA
 151  TCCTTAGGTC TTGGAGTACT TTATAGATGG TCATTATTTT TTATGATTCC
 201  TCTGAAACTA AAGGCAGAGA ATCACCCAAG CATTACAAAA ATGTTGAGCA
 251  AAATAAAAAT TAAAGCTTGG TTCCTGAACA TTATCTGTTT TCTGGTACCA
 301  CTTAAGAAGT TTCAAAGGAT GAGAAGACTA AGACTAATAA CTATTTTTTT
 351  CTTCATCTTT TCTTTCAGTG GCAGGATGTG GAAATTGATT TTTTTTTTAT
                                                       Met
 401  TTTCACCTTA ACTGAATACT TACTTAATTC TTTTGTTTAA ATTGCAGAAT
        Pro Gly Leu  Ser Cys Arg Phe Tyr Gln His Lys Phe Pro  Glu Val Glu
 451  GCCGGGTCTA AGTTGTAGAT TTTATCAACA CAAATTTCCT GAGGTGGAAG
      Asp Val Val Met Val Asn Val  Arg Ser Ile Ala Glu Met Gly Ala Tyr Val
 501  ATGTAGTGAT GGTGAATGTC AGATCCATTG CTGAAATGGG GGCTTATGTC
       Ser Leu Leu Glu Tyr Asn Asn Ile Glu Gly  Met Ile Leu Leu Ser Glu Leu
 551  AGCTTGCTGG AATACAACAA CATTGAAGGC ATGATTCTTC TTAGTGAATT
        Ala Arg Arg  Arg Ile Arg Ser Ile Asn Lys Leu Ile Arg  Ile Gly Arg
 601  AGCACGAAGG CGTATCCGTT CTATCAACAA ACTCATCCGA ATTGGCAGGA
      Asn Glu Cys Val Val Val Ile  Arg Val Asp Lys Glu Lys
 651  ATGAGTGTGT GGTTGTCATT AGGGTGGACA AAGAAAAAGG TAAGTGAGAA
 701  AAATATCTGT AATATAAATT TCAGATTTAA AATGGTTTAT TTAAAAATAC
 751  ATTTTTTGTA AATTGCAAGC TGCAGCTTAA AAAAAAAAGC TCCTTTTATA
 801  CTTAAACCTT TTACATACAA AGTTGTTAGA AAAGGATGCC AATTAGCTAT
 851  CTAAGCAAGA TCTCTTAATA GTAGTTTAAT TAGTACATCC TAGGATTTTA
 901  TGGATCAGAT AACTTGAATT TTATTTCTAG TGTTTATCAG GATCTTGATA
 951  ATTGACTCAT GGTAACCAAA CTTAGAGACA GGTAAGTCAG AGTACTAGTT
1001  CATTTACATT GGTAGGCCTG AATATGTTGG ATGCCTTTTT CTTGATTTAA
1051  AGCTAAAATA GTAATGAGAT CACTGGGTAA GCATGAAAAT GGGGCAAATG
1101  GATTTTAGGG ATCTTTTATA TAAGCTTTGA AAAAGCAATA TACTATGCGT
1151  GTATATACAC ACCTGAAATC CAACATTGTA TATCTTGCTG GTAATTAGAA
1201  TGTTTCTCGA G
```

Appendix Figure S6

Appendix Figure S6. Sequence for the eIF2α knock-in mutation. In order to generate an eIF2α non-phosphorylable cell line (U2OS RFP-LC3 eIF2αS51A), U2OS RFP-LC3 cells were co-transfected with a pX458 plasmid containing Cas9 and gRNAs to induce a DNA strand break at the appropriate site, together with a plasmid containing the DNA sequence of

replacement given here. The objective was to replace serine in position 51 by an alanine, i.e. to replace a TCC by a GCA in the gene sequence as marked in red. Moreover, to avoid further CRISPR-mediated DNA strand breaks after insertion of the knock-in sequence, additional nucleotides were exchanged without modifying the protein sequence. The corresponding amino acids of the coding sequence are indicated.

	TRANSCRIPTION			TRANSLATION		
DRUG	MEAN	SD	PVAL	MEAN	SD	PVAL
CONTROL	0	0	1	1.605149831	1.927570339	1
ALLOPURINOL	0	0	1	18.05655325	3.541557537	0.005286126
ALTRETAMINE	3.394628788	3.799518783	0.261823223	16.68488961	2.682102288	0.002049494
AMINOLEVULINIC ACID	1.813436482	1.864756618	0.23414556	29.84314879	3.544350701	0.001050216
ANASTROZOLE	1.444939972	2.502709446	0.422649731	1.865462359	3.231075586	0.911613609
ARSENIC TRIOXIDE	1.070043951	1.099597567	0.233935876	26.80346906	1.482308012	8.91472E-05
AZACITIDINE	7.920171172	2.494067482	0.031500642	14.20493735	5.661146931	0.04911728
BENDAMUSTINE HCL	12.00498106	2.090029957	0.009952663	15.00957692	5.253004828	0.035177115
BUSULFAN	12.47561556	2.41820293	0.012293452	43.78590935	1.058510423	4.56118E-05
CAPECITABINE	0	0	1	19.77429635	4.760417133	0.012558631
CARBOPLATIN	0.467709585	0.422144676	0.194986553	4.586371037	7.943827658	0.586147718
CARMUSTINE	0	0	1	0.755182882	1.308015121	0.566095029
CELECOXIB	1.171403294	2.028930022	0.422649731	17.80154202	3.211330152	0.003617172
CHLORAMBUCIL	2.910934664	3.55261988	0.291649298	28.02751826	5.428095007	0.007821395
CISPLATIN	62.74371383	2.148502041	0.000390621	69.24183252	0.621762157	1.76495E-13
CLADRIBINE	25.96279064	1.120014994	0.000619755	23.93571675	11.88085839	0.079195962
CLOFARABINE	34.55271761	5.602076353	0.072667464	36.22798114	5.272629828	0.0036289
CRIZOTINIB	14.76889075	5.187006517	0.038742819	15.59663918	2.693351614	0.002701878
CYCLOPHOSPHAMIDE	4.499610478	7.774320499	0.421698675	19.93084032	2.365820661	0.000591848
CYTARABINE HCL	20.73766941	2.608142063	0.005231221	27.6106536	2.097560927	9.80684E-05
DACARBAZINE	0	0	1	3.440281102	5.958741661	0.654425878
DACTINOMYCIN	99.62125337	0.656008415	1.44539E-05	69.84188281	4.551413264	0.000323415
DAUNORUBICIN HCL	99.3333064	1.154747182	4.50436E-05	60.81296942	5.038817843	0.000775438
DECITABINE	0	0	1	16.81537417	2.300901429	0.001064565
DEXRAZOXONE	0	0	1	18.11229036	5.197964407	0.021039476
DOCETAXEL	16.48836423	1.60214901	0.003132457	24.10326014	4.033420801	0.00377287
DOXORUBICIN HCL	95.50723329	7.781700211	0.002205548	73.14931089	3.182070525	2.79714E-05
ESTRAMUSTINE DISODIUM PHOSPHATE	0	0	1	11.94125616	13.57659891	0.317381738
ETOPOSIDE	5.7160229	1.071770703	0.011517045	0	0	0.285966809
EVEROLIMUS	14.90929683	3.428301152	0.017172056	33.32936464	3.120480939	0.000357655
EXEMESTANE	0	0	1	20.18199456	6.012057364	0.024437286
FLOXURIDINE	0	0	1	44.42586651	4.453651923	0.001016022
FLUDARABINE	1.615507472	1.849193246	0.269408016	26.68512437	0.623201572	0.000818852
FLUOROURACIL	1.901903975	3.278963145	0.420865972	15.0983535	3.743427123	0.011625063
FULVESTRANT	9.353767625	1.866993709	0.013020985	31.65003912	4.673508982	0.003228844
GEFITINIB	2.124156428	0.94025238	0.05953897	13.88203602	2.040264568	0.001648783
GEMCITABINE HCL	34.94188763	0.439327387	5.269E-05	29.80055918	5.846009345	0.008569605
HYDROXYUREA	4.19369384	5.551862208	0.320905829	12.55182133	5.257453869	0.055310899
IFOSFAMIDE	0	0	1	44.96896635	3.287701996	0.000178025
IRINOTECAN HCL	21.06256936	4.411228451	0.014307896	42.5966317	4.204234863	0.000861376
LETROZOLE	0	0	1	4.608336701	4.278902939	0.354299644
LOMUSTINE. CCNU	16.87070103	1.075880815	0.001352879	41.38445214	0.644994883	0.000251301
MEGESTROL ACETATE	0	0	1	10.72204661	3.44326966	0.025667211
MELPHALAN	0	0	1	13.73675395	1.655573604	0.001285376
MERCAPTOPURINE	8.728407706	3.226917603	0.042665498	15.6537819	5.621650534	0.038017879
METHOTREXATE	0	0	1	16.820308	2.473104196	0.001408007
METHOXSALEN	0	0	1	18.13307272	0.440585974	0.003125476
MITOMYCIN C	24.42345134	0.750288425	0.000314424	40.8414439	3.863199483	0.000625938
MITOTANE	0	0	1	12.25593713	7.889554315	0.137514185
MTX	95.65615929	4.152949759	0.000627707	99.52451732	0.823560159	1.16722E-05
OXALIPLATIN	85.38965236	1.715333453	0.000134486	79.55505616	0.453250425	9.86278E-05
PACLITAXEL	1.307029829	0.989365492	0.149356376	40.17305978	3.220040013	0.00022815
PENTOSTATIN	20.66713103	1.342020176	0.001402562	24.77950488	3.811915288	0.002696279
PLICAMYCIN	33.71635081	0.905583966	0.00024038	34.71947447	6.843760203	0.009621205
PROCARBAZINE	6.523777728	2.787050246	0.055794443	43.3708838	0.982265238	6.30661E-05
RALOXIFENE HCL	1.320946527	2.287946498	0.422649731	13.0218436	6.788374403	0.09080936
RAPAMYCIN	21.61314613	1.798152667	0.002299302	34.28836652	0.959565105	0.000141207
RESVERATROL	35.13765962	3.264691338	0.002865152	16.16381587	2.523598632	0.001792194
SPERMIDINE	17.07618773	3.513559129	0.013820223	37.4743008	5.3107741	0.003416495
STREPTOZOCIN	0.319329662	0.553095199	0.422649731	18.16747278	6.106084748	0.032892381
SUNITINIB	7.788649054	3.898172898	0.074310636	3.318085263	5.74709226	0.665000606
TAMOXIFEN CITRATE	0	0	1	19.66405781	5.287812716	0.017918265
TEMOZOLOMIDE	12.95825277	1.792875738	0.00632053	13.29933595	5.201764417	0.046567263
TENIPOSIDE	32.60854724	1.639685564	0.00084176	0.452215425	0.783260093	0.416519206
THALIDOMIDE	14.1961107	5.602271905	0.048190455	28.95102654	0.330187911	0.001265674
THIOGUANINE	9.385434192	3.686233299	0.047766231	21.04919193	2.29447502	0.000418417
THIOTEPA	25.65590567	1.648052631	0.001372623	27.45165633	1.390650295	9.27133E-05
TRETINOIN	0	0	1	0	0	0.285966809
URACIL MUSTARD	13.91279766	5.386393682	0.046505087	19.96571396	7.905500931	0.049636433
VINBLASTINE SULFATE	14.09295987	2.227025694	0.008221351	53.1195346	2.758816413	3.093E-05
VINCRISTINE SULFATE	8.323878381	3.705053351	0.060144891	39.79601469	5.504874367	0.003351568
VINORELBINE TARTRATE	3.694465409	3.763683753	0.231197094	28.71256191	1.918008565	6.60352E-05
VORINOSTAT	0	0	1	0	0	0.285966809
ZOLENDRONIC ACID	9.712809586	2.620667786	0.023417778	18.81957153	0.674921727	0.001803471

Appendix Table S1. Transcription and translation inhibition data for the custom anti-cancer library. U2OS cells were treated with a home-made library of anti-cancer agents at 3 µM for evaluating their ability to inhibit transcription (after 1.5 h treatment followed by 1 h in the presence of 5-ethynyl uridine (EU)) and translation (12 h treatment followed by 30 min in methionine-free medium and then 1.5 h in methionine-free medium supplemented with L-azidohomoalanine (AHA). The percentage of inhibition of transcription and translation was calculated and the mean of triplicates of one representative experiment among three is indicated as well as the standard deviation (SD) and the p-value (PVAL) which was calculated using a Student's t-test. These data were used to evaluate correlations with ICD parameters in **Fig. 8 and EV6**.

			Transcription inhibition		
CID	Name	Score	Mean	SD	PVAL
515328	5-Fluorodeoxycytidine	-0.073944055	0	0	0.085102669
11977753	Dactolisib	0.681357703	14.04747238	0.065312273	0.000919228
3885	β-Lapachone	-0.159663085	20.95030652	13.50085812	0.305297648
5281078	Mycophenolate mofetil	0.239570191	44.84590908	2.499182983	0.001873086
72385	Nonoxynol-9	-2.034977072	8.451037648	2.110619699	0.076812698
394347	RH-1	-0.264524221	83.36458718	3.906836417	0.006344573
101524	Becatecarin	4.967787785	69.08411747	2.477979305	0.000656291
2019	Dactinomycin	4.02340177	100	0	1.76E-07
60699	Topotecan	5.498023981	76.69808547	1.087669761	9.13E-08
372978, 108150	Trabectedin	6.127930281	100	0	1.76E-07
72271	UCN-01	4.023450825	78.94456213	1.242796208	2.13E-07

Appendix Table S2. Transcription inhibition for the positive and negative hits selected with the artificial intelligence module. Positive and negative agents were selected thanks to an algorithm that can predict ICD (**Fig. 1**). They were tested for their ability to inhibit transcription: U2OS cells were treated with these agents around their IC_{60}: 1 µM dactinomycin (DACT), 50 µM topotecan, 1 µM becatecarin, 0.5 µM trabectedin, 5 µM UCN-01, 30 µM mycophenolate mofetil, 30 µM nonoxynol-9, 25 µM dactolisib, 2.5 µM β-lapachone, 5 µM 5-fluorodeoxycytidine and 2 µM RH-1 for 1.5 h followed by 1 h where treatment was pursued in the presence of EU. The percentage of inhibition of transcription were calculated as previously described.

Nucleic acid synthesis inhibitors

CID	Name	Score
126941	METHOTREXATE	1.569
667490	MERCAPTOPURINE	-0.562
54695425	U-6591	4.316
44415057	DACTINOMYCIN	4.023
3197	C 6388	2.364
6326719	PLICAMYCIN	3.231
5746	MITOMYCIN	2.803
3657	HYDROXYUREA	2.768
3379405	THIAMIPRINE	0.571
2265	AZATHIOPRINE	-0.071
5905	IDOXURIDINE	0.028
656673	TOYOMYCIN	2.833
72511	PHLEOMYCIN	5.068
5289019	U 15167	4.988
253602	ANISOMYCIN	-0.965
3115	STALLIMYCIN	0.868
20039	2',3'-DIDEOXYADENOSINE	-0.406
4112	D-AMETHOPTERIN	1.569
54608707	FOSFONET SODIUM	4.960
284240	3'-FLUOROTHYMIDINE	-0.676
5476249	ZYGOSPORIN A	2.128
5288818	7-OMEN	5.048
47318	OLTIPRAZ	-0.689
3165	DRB	0.090
6857732	LEVOMYCIN	2.364
5718	2',3'-DIDEOXYCYTIDINE	-0.697
62999	CIPROFLOXACIN HYDROCHLORIDE	1.965
3229	1,8-NAPHTHYRIDINE-3-CARBOXYLIC ACID, 6-FLUORO-1,4-DIHYDRO-4-OXO-7-PIPERAZINYL	-0.058
4463	BI-RG-587	-0.620
5470819	CORYLIFOLIN	-1.538
4583	OFLOXACIN	0.581

Protein synthesis inhibitors

CID	Name	Score
6197	CX	-0.193
44415057	DACTINOMYCIN	4.023
439530	PUROMYCIN HYDROCHLORIDE	0.420
5959	CAM	0.427
3084037	CACTINOMYCIN	4.060
92765	CRYPTOPLEURINE	1.097
6326719	PLICAMYCIN	3.231
6603320	EMETINE	4.087
54680694	AAT 4	3.888
54712662	SANCYCLINE	6.885
5289124	U-15800	2.566
5287620	LUNATIN	-0.536
447106	SPARSOMYCIN	1.483
122806	C 1228	2.593
253602	ANISOMYCIN	-0.965
5354042	VERNAMYCIN A	0.649
278679	CEPHALOTAXINE	1.009
285033	HOMOHARRINGTONINE	2.730
122731	STAPHYLOMYCIN S	1.132
99558	2-(AMINOETHYL)CYSTEINE MONOHYDROCHLORIDE	1.911
429598	BOUVARDIN	2.999
9553856	LEVOFURALTADONE	-1.044
5459220	KRN5500	1.467
400771	STENOROL	2.981
439647	TPCK	-0.769

Antimetabolites

CID	Name	Score
126941	METHOTREXATE	1.569
2723601	THIOGUANINE	0.477
667490	MERCAPTOPURINE	-0.562
75420	4 APP	-0.571
24180949	SODIUM PHENYLACETATE	1.117
439530	PUROMYCIN HYDROCHLORIDE	0.420
9048	NSC 3074	2.909
676166	6-MPR	0.537
222284	HARZOL	0.759
71558	NICOTINIC ACID	2.127
3385	FLUOROURACIL	-0.384
5790	FLOXURIDINE	-0.201
3938248	TGS	1.170
236184	BDU	-0.190
3379405	THIAMIPRINE	0.571
2265	AZATHIOPRINE	-0.071
5905	IDOXURIDINE	0.028
247955	B181008	1.025
5351180	CYTARABINE HYDROCHLORIDE	3.542
2796	CLOFIBRATE	-1.265
4912	PROBUCOL	0.645
25674	D-ETHIONINE	1.113
20039	2',3'-DIDEOXYADENOSINE	-0.406
9444	AZACITIDINE	0.613
3366	FLUCYTOSINE	-0.165
4112	D-AMETHOPTERIN	1.569
999	BENZENACETIC ACID	-1.024
16886	DECITABINE	0.114
284240	3'-FLUOROTHYMIDINE	-0.676
25050	NSC-145668	3.374
5386	FT 207	-1.256
5064	RIBAVIRIN	0.580
294641	ACIA	0.363
3034016	AAFC	0.238
1651	3DADO	0.472
39981	NA	2.283
55710	ICN 4221	0.132
125219	D 19391	-0.768
457954	TIAZOFURIN	0.825
596	CYTARABINE	0.306
5694	WY-14,643	-0.459
30751	FLUDARABINE PHOSPHATE	1.344
21704	ARA-A	0.587
3599	MILTEFOSIN C	-0.707
5718	2',3'-DIDEOXYCYTIDINE	-0.697
5353599	(E)-5-(2-BROMOVINYL)-2'-DEOXYURIDINE	-0.097
53232	MEVACOR	-0.135
54454	SIMVASTATIN	-0.057
148177	D 21266	-1.659

PARP inhibitors

CID	Name	Score
1645	M-AMINOBENZAMIDE	0.020
1853	6-PHENANTHRIDONE	-1.140
11960529	ABT-888	0.143
23725625	OLAPARIB	-0.297

Table 3

Appendix Table S3. ICD scores of specific categories of agents. The 50,000 compounds of the NCI-60 library were annotated for different parameters including nucleic acid synthesis inhibition and protein synthesis inhibition. The predicted ICD score of agents from these categories (score) was calculated with a previously described artificial intelligence model. The name and compound ID number (CID) are indicated. Two other random categories were chosen as controls: PARP inhibitors and antimetabolites (n=49); their predicted ICD score were also calculated with this artificial intelligence model.

Cell line construction	Name	Sequence	Purpose	
eIF2αS51A	474CRISPRoligo_Hh-chr14_S	CACCGTTCTTAGTGAATTATCCAGA	sgRNA to insert into a CRISPR-Cas9 vector (pX458) for eIF2α knock-in	A
	475CRISPRoligo_Hh-chr14_AS	AAACTCTGGATAATTCACTAAGAAC		
	478EIF2A_seqF	GTGGCAGGATGTGGAAATTGATT	primers to perform PCR to amplify eIF2α mutated region before sending for sequencing	B
	479EIF2A_seqR	TCTCACTTACCTTTTTCTTTGTCCAC (gtggacaaagaaaaaggtaagtgaga)		
	482EIF2A_seq	AGAATGCCGGGTCTAAGTTGT	to sequence mutated eIF2α region	C
eIF2AK1-/-	K1 gRNA	TATTCGGGGTCCGGGCCTCGG	gRNAs in a U6gRNA-Cas9-2A-RFP (Sigma-Aldrich)	D
eIF2AK2-/-	K2 gRNA	TTCAGGACCTCCACATGATAGG		
eIF2AK3-/-	K3 gRNA	AGGTATATCTGTTCAGCTCTGG		
eIF2AK4-/-	K4 gRNA	GAACGGCTGCCATTCTACATGG		

Appendix Table S4. DNA sequences for U2OS eIF2αS51A and eIF2AK-/- generation. To construct the eIF2α non-phosphorylable cell line (U2OS RFP-LC3 eIF2αS51A), U2OS RFP-LC3 cells were co-transfected with a homology repair oligo coding for the knock-in mutation (**Fig. S4**) together with a pX458 plasmid containing Cas9 and specific gRNAs whose sequences

are given (**A**). After transfection, the DNA of cells that were able to proliferate was collected and amplified by PCR (**B**) before sending them for sequencing (**C**). To construct the eIF2α kinases knock out cell lines (U2OS GFP-LC3 eIF2AK1$^{-/-}$, eIF2AK2$^{-/-}$, eIF2AK3$^{-/-}$ and eIF2AK4$^{-/-}$), cells were transected with all-in one plasmids from Sigma-Aldrich containing Cas9 and the indicated gRNAs (**D**).

**Figure 2**	**Groups**		**p value**
**C) CALR, video IF**	MTX	Ctr	1,50E-05
Mean +/- SD of triplicate from 1 representative exp. among 3	DACT 0.5	Ctr	1,10E-05
Student's t-test (R)	DACT 1	Ctr	8,90E-03
**F) HMGB1, video IF**	MTX	Ctr	6,80E-03
Mean +/- SD of quadruplicate from 1 representative exp. among 3	DACT 0.5	Ctr	2,20E-02
Student's t-test (R)	DACT 1	Ctr	4,70E-04
**H) ATP, IF**	MTX 6h	Ctr 6h	0,00015333
Mean +/- SD of quadruplicate from 1 representative exp. among 3	MTX 12h	Ctr 12h	8,22E-06
Student's t-test (R)	MTX 24h	Ctr 24h	1,65E-06
	DACT 0.5 6h	Ctr 6h	0,001476146
	DACT 0.5 12h	Ctr 12h	0,083606433
	DACT 0.5 24h	Ctr 24h	0,000601737
	DACT 1 6h	Ctr 6h	0,014935182
	DACT 1 12h	Ctr 12h	0,001252852
	DACT 1 24h	Ctr 24h	0,000102997
**J) IFN1, IF**	MTX	Ctr	3,48E-09
Mean +/-SEM of 5 independent exp.	IFNα1	Ctr	5,58E-06
Student's t-test (R)	DACT 0.5	Ctr	4,54E-05
	DACT 1	Ctr	1,14E-03
**L) CALR, Cytometry**	MTX	Ctr	0,046972592
Mean +/-SEM of 6 independent experiments	DACT 0.5	Ctr	0,047746877
Student's t-test (Excel)	DACT 1	Ctr	0,026430706
**M) HMGB1, ELISA**	MTX	Ctr	0,041171619
Mean +/-SEM of 4 independent experiments	DACT 0.5	Ctr	0,043039251
Student's t-test (Excel)	DACT 1	Ctr	0,041743204
**N) ATP, BioluminescenceAssay**	MTX	Ctr	0,032937717
Mean +/- SD of triplicate from 1 representative exp. among 3	DACT 0.5	Ctr	0,001542082

Student's t-test (Excel)	DACT 1	Ctr	0,002351196

**Figure 3**	**Groups**		**p value**
**B) peIF2α, IF**	THAPS	Ctr	0,029149288
Mean +/-SEM of 3 independent exp.	DACT 0.25	Ctr	0,003120227
Student's t-test (R)	DACT 0.5	Ctr	0,018061545
	DACT 1	Ctr	0,013543452
**D) ATF4, IF**	THAPS	Ctr	0,01344063
Mean +/-SEM of 5 independent exp.	DACT 0.25	Ctr	0,12845239
Student's t-test (R)	DACT 0.5	Ctr	0,05701633
	DACT 1	Ctr	0,08760908
**F) ATF6, IF**	THAPS	Ctr	1,05E-04
Mean +/-SEM of 6 independent exp.	DACT 0.25	Ctr	8,87E-01
Student's t-test (R)	DACT 0.5	Ctr	5,32E-01
	DACT 1	Ctr	5,19E-02
**H) XBP1, IF**	THAPS	Ctr	0,000217507
Mean +/-SEM of 5 independent exp.	DACT 0.25	Ctr	0,753570158
Student's t-test (R)	DACT 0.5	Ctr	0,644922089
	DACT 1	Ctr	0,773716594
**J) peIF2α kinases, IF**	THAPS WT	Ctr WT	0,000952716
Mean +/-SEM of 3 independent exp.	DACT WT	Ctr WT	0,008865305
Pairwise multiple comparisons test (R)	THAPS K1	Ctr K1	0,000159565
	DACT K1	Ctr K1	0,156766864
	THAPS K2	Ctr K2	0,001798423
	DACT K2	Ctr K2	0,067468508
	THAPS K3	Ctr K3	0,356877726
	DACT K3	Ctr K3	0,755373822
	THAPS K4	Ctr K4	6,75E-07
	DACT K4	Ctr K4	0,001798423
	THAPS K1	THAPS WT	0,641458396
	THAPS K2	THAPS WT	0,156766864
	THAPS K3	THAPS WT	0,000597212
	THAPS K4	THAPS WT	0,156766864
	DACT K1	DACT WT	0,213167232
	DACT K2	DACT WT	0,432893435
	DACT K3	DACT WT	0,003644329
	DACT K4	DACT WT	0,594443002
**L) peIF2α ex vivo, IF**	TM (5)	Ctr (8)	0,021795268
Mean +/-SD of each mouse data	DACT 6h (8)	Ctr (8)	0,008385681
Student's t-test (R)	DACT 24h (6)	Ctr (8)	0,869451002

**N) CALR ex vivo, IF**<br>Mean +/-SD of each mouse data<br>Student's t-test (R)	TM (5)	Ctr (8)	0,360972484
	DACT 6h (8)	Ctr (8)	0,077518631
	DACT 24h (6)	Ctr (8)	0,002390871

**Figure 4**	**Groups**		**LogRank p value**
**B) Phagocytosis**<br>Mean +/-SEM of 3 independent exp.<br>Pairwise multiple comparisons test (R)	Ctr 37°C	Ctr 4°C	0,955200917
	OXA 37°C	OXA 4°C	0,008756406
	DACT 37°C	DACT 4°C	0,008756406
	OXA 4°C	Ctr 4°C	0,256260458
	DACT 4°C	Ctr 4°C	0,136501018
	OXA 37°C	Ctr 37°C	0,00142704
	DACT 37°C	Ctr 37°C	0,001125467
**C) MHCII**<br>Mean +/-SEM of 5 independent exp.<br>Student's t-test (Excel)	LPS+IFNγ	Ctr	0,002
	OXA	Ctr	0,0031
	DACT 0.25	Ctr	<0.0001
	DACT 0.5	Ctr	<0.0001
	DACT 1	Ctr	0,0003
**D) CD86**<br>Mean +/-SEM of 5 independent exp.<br>Student's t-test (Excel)	LPS+IFNγ	Ctr	<0.0001
	OXA	Ctr	0,0105
	DACT 0.25	Ctr	0,0023
	DACT 0.5	Ctr	0,0023
	DACT 1	Ctr	0,0101
**G) Vaccination-Survival**<br>Survival of 1 representative exp. among 3<br>LogRank test (TumGrowth software package)	DACT (7)	Ctr (8)	0,0002

**Figure 5**	**Groups**		**Mean tumor growth**<br>Type II ANOVA (TumGrowth software package)<br>**p value**	**Survival**<br>LogRank test (TumGrowth software package)<br>**LogRank p value**
**D, E, G, H) In vivo effect of DACT +/- aPD-1 +/- aCD4/aCD8**	DACT (8)	Ctr (9)	<0.0001	0,0075
	DACT+aCD4/aCD8 (9)	aCD4/aCD8 (9)	0,4488	0,1239
	DACT+aCD4/aCD8 (9)	DACT (8)	<0.0001	<0.0001
	aCD4/aCD8 (9)	Ctr (9)	0,0119	0,0049
	DACT+aPD-1 (7)	aPD-1 (8)	<0.0001	0,0013
	DACT+aPD-1 (7)	DACT (8)	0,2566	0,2741
	DACT+aPD-1 (7)	Ctr (9)	<0.0001	0,0013

	aPD-1 (8)	Ctr (9)	0,3021	0,509

			Survival
			LogRank test (TumGrowth software package)
			**LogRank p value**
**K) Rechallenge**	Cured (11)	Naïve (5)	<0.0001

**Figure 6**	**Groups**		**p-value**
**B) IFNγ, qPCR**<br>Mean +/- of each mouse data from 2 independent exp.<br><br>Student's t-test (GraphPad)	DACT (23)	Ctr (22)	0,0259

	**Groups**		**Mean tumor growth**	**Survival**
			Type II ANOVA (TumGrowth software package)	LogRank test (TumGrowth software package)
			**p value**	**LogRank p value**
**E, F) In vivo effect of DACT +/- aIFNγ**	DACT (8)	Ctr (7)	0,0419	0,0152
	DACT+aIFNγ (8)	aIFNγ (7)	0,1235	0,1734
	DACT+aIFNγ (8)	DACT (8)	0,3592	0,4676
	aIFNγ (7)	Ctr (7)	0,7349	0,926

**Figure 7**		**B) Transcription inhibition, EU**<br><br>Mean +/- SD of quadruplicate from 1 representative exp. among 3<br><br>Student's t-test (R)	**D) Transcription inhibition, FBL-NCL**<br><br>Mean +/- SD of quadruplicate from 1 representative exp. among 3<br><br>Student's t-test (R)	**F) Translation inhibition, AHA**<br><br>Mean +/- SD of quadruplicate from 1 representative exp. among 3<br><br>Student's t-test (R)
**Groups**		**p value**		
DACT 0.5	Ctr	2,13E-16	9.78235981592678e-10	3,49E-06
DACT 1	Ctr	1,38E-17	2.484857480609e-16	1,27E-08
DACT 5	Ctr	6,73E-17	8.07113378087854e-06	3,36E-08
BTZ 0.5	Ctr	2,12E-01	2.94507283335182e-06	5,29E-11
BTZ 1	Ctr	1,91E-01	9.55683085110637e-07	7,09E-11
BTZ 5	Ctr	1,97E-02	0.00406981296887172	5,22E-10
CDDP 75	Ctr	2,81E-01	0.00251764516117751	1,36E-03
CDDP 150	Ctr	9,89E-06	0.00147438328122473	8,03E-07

CDDP 300	Ctr	4,00E-04	4.85975742999306e-06	1,06E-04
CRIZO 10	Ctr	7,66E-13	0.00622805916226957	6,52E-03
CRIZO 20	Ctr	1,20E-03	0.000636415429439258	1,51E-04
CRIZO 40	Ctr	5,16E-07	1.59936200125524e-09	1,88E-09
DAUNO 0.5	Ctr	9,46E-15	5.56641355469102e-16	3,39E-08
DAUNO 1	Ctr	3,33E-07	8.3087695351635e-08	1,16E-10
DAUNO 5	Ctr	1,40E-16	7.43217205947045e-13	1,91E-09
DOC 0.5	Ctr	4,36E-02	0.337287264492058	4,20E-01
DOC 1	Ctr	4,36E-02	0.14910057616936	4,34E-01
DOC 5	Ctr	4,36E-02	0.218242188471649	2,44E-05
DOXO 0.5	Ctr	1,01E-12	2.06322981656859e-05	9,83E-03
DOXO 1	Ctr	5,11E-09	1.54998027427469e-06	5,20E-06
DOXO 5	Ctr	6,79E-17	1.43382046996478e-05	6,59E-07
EPI 0.5	Ctr	8,17E-13	8.23169101113265e-06	2,98E-01
EPI 1	Ctr	1,49E-04	1.45309610655136e-17	6,57E-01
EPI 5	Ctr	8,05E-16	3.95502749477921e-05	2,82E-03
MTX 0.5	Ctr	1,54E-13	6.7638958979284e-14	2,98E-01
MTX 1	Ctr	5,54E-04	6.7638958979284e-14	5,17E-03
MTX 5	Ctr	2,69E-16	6.7638958979284e-14	9,39E-10
OXA 250	Ctr	4,09E-04	3.92253643651811e-06	1,42E-09
OXA 500	Ctr	1,50E-04	1.55874864708109e-07	1,70E-06
OXA 1000	Ctr	2,04E-04	7.56740516085633e-06	2,36E-11
PACL 0.5	Ctr	4,36E-02	0.00188584778105787	3,77E-05
PACL 1	Ctr	4,36E-02	0.0903964518384545	2,97E-03
PACL 5	Ctr	4,36E-02	0.599244580147299	2,40E-02
VB 0.5	Ctr	4,36E-02	0.00666383224561728	5,77E-09
VB 1	Ctr	2,42E-01	0.000247806243761579	8,33E-09
VB 5	Ctr	1,39E-01	0.00031167957216998	3,13E-06
VC 0.5	Ctr	4,36E-02	0.0101023161356121	3,48E-08
VC 1	Ctr	4,36E-02	0.000361387990917593	5,47E-10
VC 5	Ctr	8,31E-02	0.000489599872636203	2,56E-08

**Figure EV2**	**Groups**		**p value**
**A) peIF2α, WB** Mean +/-SEM of 4 independent exp. Student's t-test (R)	THAPS	Ctr	0,046740273
	DACT 0.5	Ctr	0,002562029
	DACT 1	Ctr	0,006155752
	DACT 2	Ctr	0,004986772
**C) CALR, Cytometry** Mean +/-SEM of 6 independent exp. Student's t-test (Excel)	MTX	Ctr	0,026879887
	DACT 0.5	Ctr	0,00848546
	DACT 1	Ctr	0,004918971
**D) ATP, Bioluminescence assay**	MTX	Ctr	0,005921447

Mean +/- SD of triplicate from 1 representative exp. among 3	DACT 0.5	Ctr	0,000428236
Student's t-test (Excel)	DACT 1	Ctr	2,71625E-06
**E) HMGB1, ELISA**	MTX	Ctr	0,015940497
Mean +/-SEM of 5 independent exp.	DACT 0.5	Ctr	0,012630298
Student's t-test (Excel)	DACT 1	Ctr	0,025597172
**G) IFN1, IF**	MTX	Ctr	2,86E-07
Mean +/-SEM of 5 independent exp.	IFNα1	Ctr	3,12E-03
Student's t-test (R)	DACT 0.5	Ctr	5,99E-04
	DACT 1	Ctr	2,45E-02

**Figure EV3**	**Groups**		**Mean tumor growth**	**Survival**
			Type II ANOVA (TumGrowth software package)	LogRank test (TumGrowth software package)
			**p value**	**LogRank p value**
**D, E) In vivo effect of DACT+CDDP in C57Bl/6 mice**	DACT (10)	Ctr (9)	0,3354	0,0008
	CDDP (9)	Ctr (9)	0,4116	0,7881
	MTX (8)	Ctr (9)	0,0056	0,0045
	DACT+CDDP (10)	CDDP (9)	0,0003	<0.0001
	DACT+CDDP (10)	DACT (10)	0,0355	0,0022
	DACT+CDDP (10)	Ctr (9)	0,0039	<0.0001
**I, J) In vivo effect of DACT+CDDP in Nude mice**	DACT (10)	Ctr (10)	0,356	0,0628
	CDDP (9)	Ctr (10)	0,3927	0,5191
	MTX (10)	Ctr (10)	0,8284	0,5916
	DACT+CDDP (10)	CDDP (9)	0,9283	0,2783
	DACT+CDDP (10)	DACT (10)	0,0905	0,838
	DACT+CDDP (10)	Ctr (10)	0,4054	0,1281
**N, O) In vivo effect of DACT+CDDP+aPD-1**	DACT (10)	Ctr (9)	0,2167	0,0077
	CDDP (10)	Ctr (9)	0,9984	0,9931
	aPD-1 (9)	Ctr (9)	0,0662	0,0066
	DACT+CDDP (10)	CDDP (10)	0,2712	0,0204
	DACT+CDDP (10)	DACT (10)	0,8573	0,7607

	DACT+CDDP (10)	Ctr (9)	0,287	0,0268
	DACT+aPD-1 (10)	DACT (10)	0,0057	0,0001
	DACT+aPD-1 (10)	aPD-1 (9)	0,0393	0,0183
	DACT+aPD-1 (10)	Ctr (9)	0,0002	<0.0001
	CDDP+aPD-1 (9)	CDDP (10)	0,0734	0,0344
	CDDP+aPD-1 (9)	aPD-1 (9)	0,9189	0,7594
	CDDP+aPD-1 (9)	Ctr (9)	0,0824	0,0356
	DACT+CDDP+aPD-1 (9)	DACT+CDDP (10)	0,0006	0,0037
	DACT+CDDP+aPD-1 (9)	DACT+aPD-1 (10)	0,5014	0,4479
	DACT+CDDP+aPD-1 (9)	CDDP+aPD-1 (9)	0,0067	0,0135
	DACT+CDDP+aPD-1 (9)	DACT (10)	0,001	0,0052
	DACT+CDDP+aPD-1 (9)	CDDP (10)	<0.0001	0,0002
	DACT+CDDP+aPD-1 (9)	aPD-1 (9)	0,0088	0,0252
	DACT+CDDP+aPD-1 (9)	Ctr (9)	<0.0001	0,0002

			**Survival**
			Type II ANOVA (TumGrowth software package)
			**p value**
**P, Q) Rechallenge**	Cured (4)	Naïve (5)	0,0005

**Figure EV4**		**B) CD8$^+$/CD4$^+$FoxP3$^+$** Student's t-test (GraphPad)
**Groups**		**p-value**
DACT (10)	Ctr (10)	0,041
		**C) CD3$^-$NK1.1$^+$**

Student's t-test (GraphPad)
**p-value**
0,0305
**D) CD3$^+$NK1.1$^+$** Student's t-test (GraphPad)
**p-value**
0,0222
**E) IL17 in CD4$^+$CD8$^-$** Student's t-test (GraphPad)
**p-value**
0,0068
**F) IL17 in CD4$^-$CD8$^+$** Student's t-test (GraphPad)
**p-value**
0,0243
**G) IL17 in CD3$^+$TCRgd$^+$** Student's t-test (GraphPad)
**p-value**
0,0721
**H) IFNγ in CD4$^-$CD8$^+$** Student's t-test (GraphPad)
**p-value**
0,1265
**I) IL4 in CD4$^+$CD8$^-$** Student's t-test (GraphPad)
**p-value**
0,4646

**Figure EV5**	**B) Translation inhibition, AHA**

			Mean +/- SD of quadruplicate from 1 representative exp. among 3<br><br>Student's t-test (R)
Groups			
WT	CHX	Ctr	8,00E-31
	THAPS	Ctr	3,26E-31
	DACT	Ctr	4,22E-18
	FLAVO	Ctr	5,65E-25
	LURBI	Ctr	7,90E-16
	BTZ	Ctr	1,85E-20
	CDDP	Ctr	8,29E-31
	CRIZ	Ctr	2,91E-25
	DAUN	Ctr	2,34E-31
	DOC	Ctr	3,66E-06
	DOXO	Ctr	1,78E-31
	EPI	Ctr	5,22E-28
	MTX	Ctr	1,78E-31
	OXA	Ctr	3,52E-28
	PACL	Ctr	6,51E-05
	VB	Ctr	9,73E-11
	VC	Ctr	5,01E-11
	Ctr ISRIB	Ctr WT	0,925516588
	Ctr eIF2aS51A	Ctr WT	0,039781052
	CHX ISRIB	CHX WT	0,935088337
	CHX eIF2αS51A	CHX WT	0,708190897
	THAPS ISRIB	THAPS WT	4,36E-10
	THAPS eIF2aS51A	THAPS WT	1,07E-20
	DACT ISRIB	DACT WT	0,10764557
	DACT eIF2aS51A	DACT WT	0,022295146
	FLAVO ISRIB	FLAVO WT	0,697041909
	FLAVO eIF2aS51A	FLAVO WT	0,225823573
	LURBI ISRIB	LURBI WT	0,70804377
	LURBI eIF2aS51A	LURBI WT	0,01346988
	BTZ ISRIB	BTZ WT	0,164902683
	BTZ eIF2aS51A	BTZ WT	0,159869469
	CDDP ISRIB	CDDP WT	0,453742982

CDDP eIF2aS51A	CDDP WT	0,99590432
CRIZ ISRIB	CRIZ WT	0,021604526
CRIZ eIF2aS51A	CRIZ WT	0,271658825
DAUN ISRIB	DAUN WT	0,84542227
DAUN eIF2aS51A	DAUN WT	1
DOC ISRIB	DOC WT	0,667754708
DOC eIF2aS51A	DOC WT	0,402779966
DOXO ISRIB	DOXO WT	1
DOXO eIF2aS51A	DOXO WT	1
EPI ISRIB	EPI WT	0,554909639
EPI eIF2aS51A	EPI WT	0,444244333
MTX ISRIB	MTX WT	1
MTX eIF2aS51A	MTX WT	1
OXA ISRIB	OXA WT	0,649105822
OXA eIF2aS51A	OXA WT	0,470368039
PACL ISRIB	PACL WT	0,971150986
PACL eIF2aS51A	PACL WT	0,371177791
VB ISRIB	VB WT	0,833967549
VB eIF2aS51A	VB WT	0,749286903
VC ISRIB	VC WT	0,401817745
VC eIF2aS51A	VC WT	0,090095054

		**F) Percentage GFP inhibition**<br><br>Mean +/- SD 2 experiments - Statistics of 3 experiments<br><br>Student's t-test (R)
		**p-value**
DACT	Ctr	2.3e−03
CRIZ	Ctr	2.3e−03
DAUN	Ctr	1.4e−02
DOXO	Ctr	8.6e−03
EPI	Ctr	6.8e−05
LURBI	Ctr	2.2e−03
MTX	Ctr	1.8e−04
OXA	Ctr	6.9e−05
		**G) Percentage reversibility**<br><br>Mean +/- SD 2 experiments - Statistics of 3 experiments

		Student's t-test (R)
		**p-value**
DACT	Ctr	2.1e−01
CRIZ	Ctr	2.3e−03
DAUN	Ctr	1.8e−01
DOXO	Ctr	1
EPI	Ctr	2.1e−01
LURBI	Ctr	2.1e−01
MTX	Ctr	4.6e−02
OXA	Ctr	1.1e−02

Appendix Table S5. Statistical tests and p-values. Statistical test and calculated p-values as reported in the manuscript.
