# Supplementary material for: Inhibition of transcription by dactinomycin reveals a new characteristic of immunogenic cell stress
Source: EMBO Mol Med. 2020 Apr 23;12(5):e11622. doi: 10.15252/emmm.201911622 (PMC7207166; doi:10.15252/emmm.201911622)
Supplement: Supplementary file 2 — Expanded View Figures PDF [file EMMM-12-e11622-s002.pdf]

Expanded View Figures

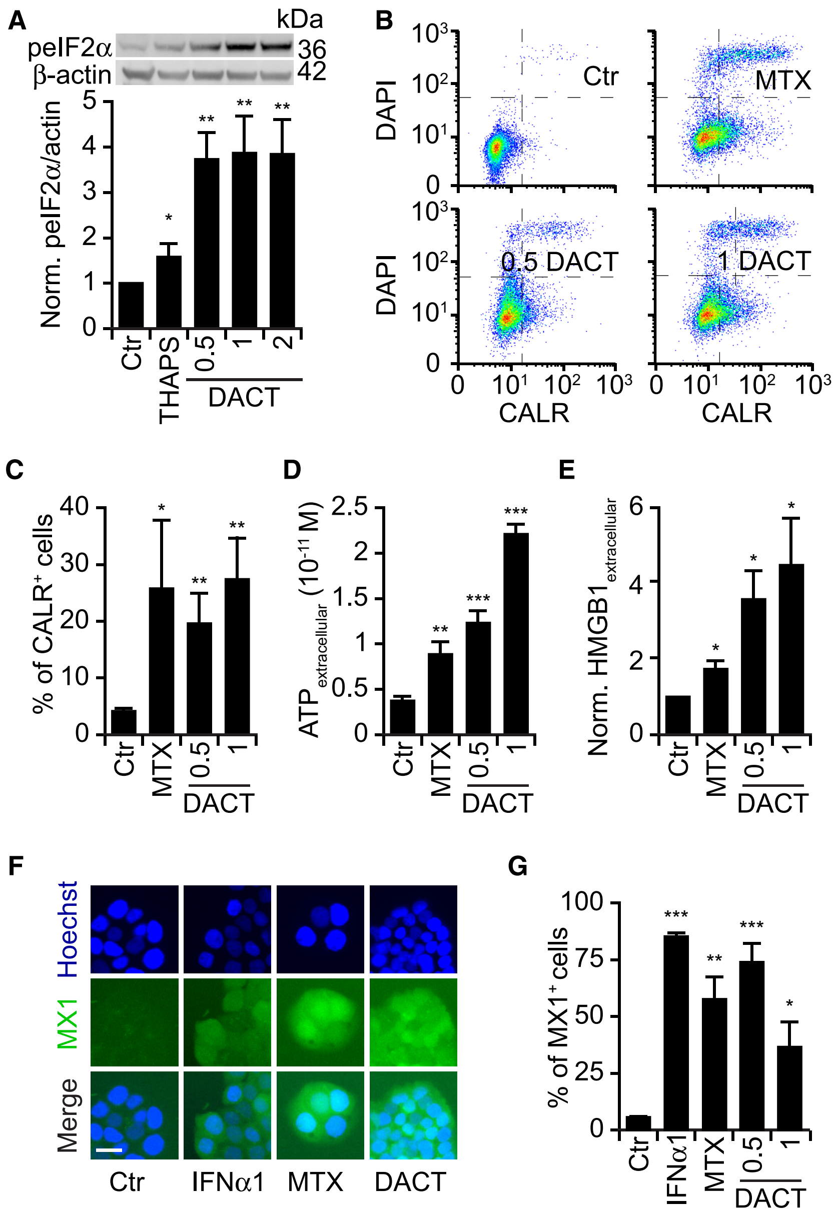

Figure EV1. Immunogenic cell death hallmarks in mouse cancer cells.

A Mouse fibrosarcoma MCA205 cells were treated with 0.5, 1, or 2 μM dactinomycin (DACT) for 6 h. Thapsigargin (THAPS) at 3 μM was used as a positive control. Cells were harvested and subjected to SDS–PAGE. The phosphorylation of eIF2α was quantified by means of phosphoneoepitope-specific eIF2α antibody. Barcharts of means ± SEM of peIF2α intensity normalized with β-actin intensity of four independent immunoblots are depicted, and representative images from one experiment are shown.

B, C MCA205 cells were treated with dactinomycin (DACT) at a concentration of 0.5 and 1 μM. Mitoxantrone (MTX) was used at 4 μM as a positive control. Cells were treated with these for 6 h, and then, medium was refreshed. Twenty-four h later, cells were collected and surface-exposed calreticulin (CALR) was stained with an antibody specific for CALR. DAPI was used as an exclusion dye, and cells were acquired by flow cytometry. The mean ± SEM of the percentage of $CALR^+$ cells among viable ($DAPI^-$) ones in six independent experiments is depicted.

D MCA205 cells were treated as described above for 24 h. Concentration of secreted ATP in the supernatants was quantified with a luciferase-based bioluminescence kit. The mean ± SD of one representative experiment among three is shown.

E MCA205 cells were treated as described above for 24 h, and the concentration of HMGB1 in the supernatants was quantified with an ELISA kit. The mean ± SEM of four independent experiments is depicted.

F, G MCA205 cells were treated as described above for 6 h. Then, medium was changed and 24 h later, type I interferon response was assessed by transferring the supernatant on HT29 MX1-GFP reporter cells lines cells for additional 48 h. Some cells were incubated with interferon α1 (IFNα1) for 48 h as an additional control. Images were acquired by fluorescence microscopy; representative images of Ctr, IFNα1, MTX, and DACT 1 μM are shown (F). Scale bar represents 20 μm. The number of positive cells was assessed based on the distribution of cellular green fluorescence intensity in IFNα1 versus control (Ctr) conditions. The mean ± SEM of five independent experiments is depicted (G).

Data information: *P*-values indicating statistically significant differences to the control were calculated with Student's *t*-test: $^*P < 0.05$, $^{**}P < 0.01$, $^{***}P < 0.001$ (A, C, D, E, G).

© 2020 The Authors

Figure EV2. DACT sensitizes MCA205 to CDDP in an immune-dependent manner and to immunotherapy.

A–J 3×10^5 mouse fibrosarcoma MCA205 cells were injected subcutaneously (*s.c*) into the flank of immunocompetent syngeneic C57Bl/6 mice with *n* mice per group ($n = 8$ for MTX and CDDP, $n = 9$ for Ctr, or $n = 10$ for DACT and CDDP + DACT) (A-E) or athymic immunodeficient *nu/nu* mice ($n = 9$ for CDDP and $n = 10$ for other groups) (F-J). When tumors became palpable, the mice were treated intraperitoneally (*i.p.*) with injectable solution (Ctr), 5.17 mg/kg mitoxantrone (MTX), 0.5 mg/kg cisplatin (CDDP), 0.5 mg/kg dactinomycin (DACT), or the combination of CDDP + DACT. Tumor size was assessed regularly, and tumor growth of DACT versus controls Ctr (B, G) and DACT + CDDP versus CDDP alone (C, H) is depicted. Mean tumor area for each group was calculated, and significances were tested using a type II ANOVA test (D, I). Overall survival is depicted, and *P*-values were calculated with a log-rank test (E, J). Stars indicate the *P*-value of each treatment versus Ctr control, and hashes indicate *P*-values of the combination treatment versus CDDP alone ($^{**/\#\#}P < 0.01$, $^{***/\#\#\#}P < 0.001$) (D, E).

K–Q 1×10^5 MCA205 cells were injected subcutaneously (*s.c.*) into the flank of immunocompetent syngeneic C57Bl/6 mice ($n = 9$ for Ctr, Ctr + anti-PD-1, CDDP + anti-PD-1, and CDDP + DACT + anti-PD-1; $n = 10$ for CDDP, DACT, DACT + anti-PD-1, CDDP + DACT) (K). When tumors became palpable, mice were treated intraperitoneally (*i.p*) with injectable solution, 0.5 mg/kg CDDP, 0.5 mg/kg DACT, or the combination of CDDP + DACT. At days 6, 10, and 14 after chemotherapy, mice were treated *i.p.* with 100 μg anti-PD-1 per mouse or a corresponding isotype. Tumor size was assessed regularly, and tumor growth of DACT + PD-1 versus PD-1 alone (L) and DACT + CDDP + PD-1 versus CDDP + PD-1 (M) is shown. Mean tumor area for each group was calculated, and significances were determined using a type II ANOVA test. (N). Overall survival is depicted, and *P*-values were calculated with a log-rank test (O). * indicates the *P*-value of DACT + anti-PD-1 or CDDP + DACT + anti-PD-1 versus the respective conditions without anti-PD-1; # indicates the *P*-values of DACT + anti-PD-1 or CDDP + DACT+anti-PD-1 compared to the respective conditions without DACT ($^{*/\#}P < 0.05$, $^{**/\#\#}P < 0.01$, $^{***/\#\#\#}P < 0.001$) (N, O). The four surviving mice from this experiment were rechallenged with MCA205 and TC-1 cells injected in opposite flanks. Five naïve mice were co-injected as controls. Individual growth curves of both MCA205 and TC-1 tumors are depicted for cured (P) and naïve mice (Q). The tumor area formed by MCA205 growth in cured mice was compared to the one in naïve mice was calculated using a type II ANOVA test ($^{***}P < 0.001$) (Q).

▶

 © 2020 The Authors

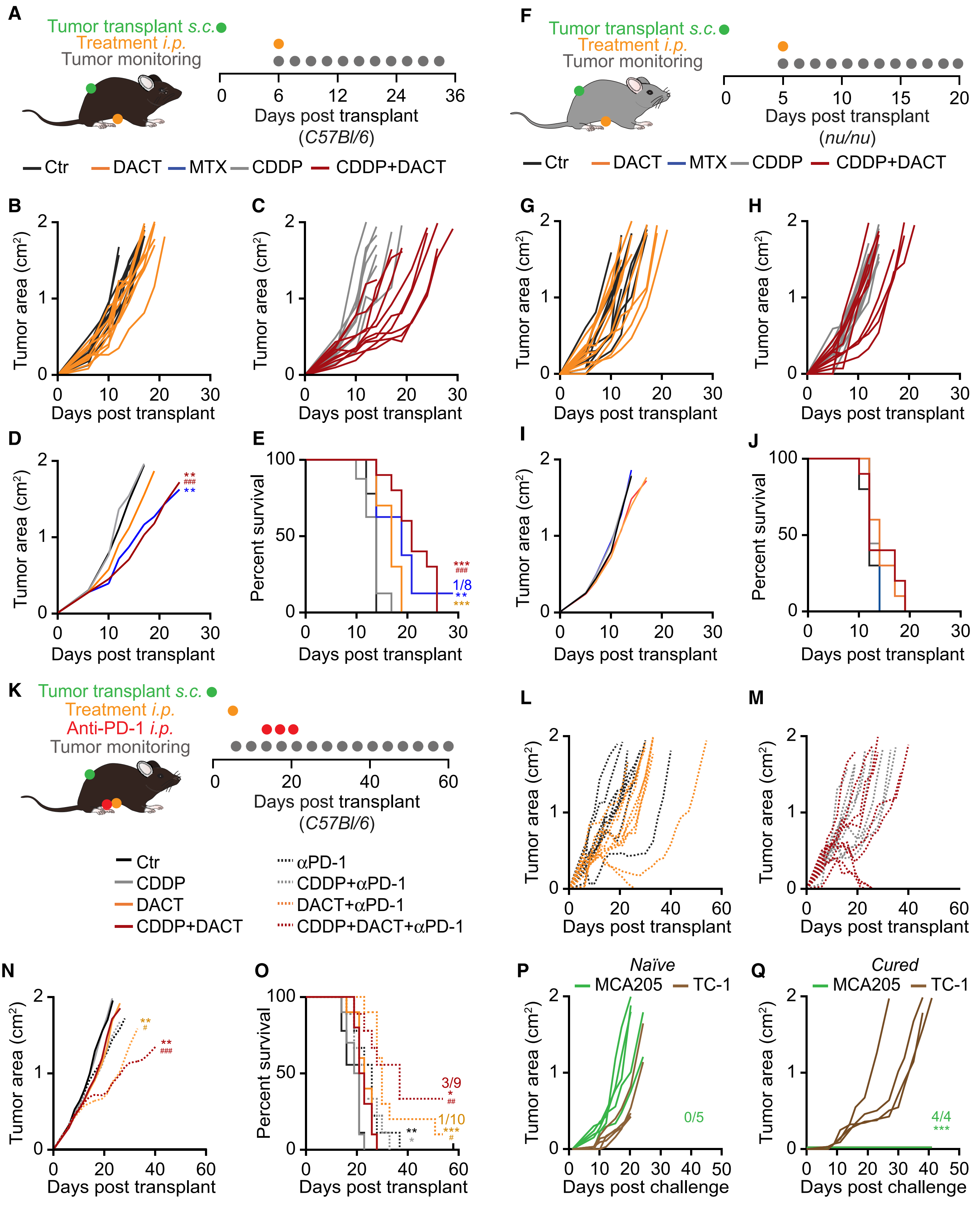

A
Tumor transplant s.c.
Treatment i.p.
Tumor monitoring
0 6 12 24 36
Days post transplant
(C57Bl/6)
Ctr DACT MTX CDDP CDDP+DACT
F
Tumor transplant s.c.
Treatment i.p.
Tumor monitoring
0 5 10 15 20
Days post transplant
(nu/nu)
Ctr DACT MTX CDDP CDDP+DACT
B C G H
Tumor area (cm²)
Days post transplant
D E I J
Percent survival
1/8
K
Tumor transplant s.c.
Treatment i.p.
Anti-PD-1 i.p.
Tumor monitoring
0 20 40 60
Days post transplant
(C57Bl/6)
Ctr
CDDP
DACT
CDDP+DACT
αPD-1
CDDP+αPD-1
DACT+αPD-1
CDDP+DACT+αPD-1
L M N O
3/9
1/10
P
Naïve
MCA205 TC-1
0/5
Days post challenge
Q
Cured
MCA205 TC-1
4/4
Days post challenge

Figure EV2.

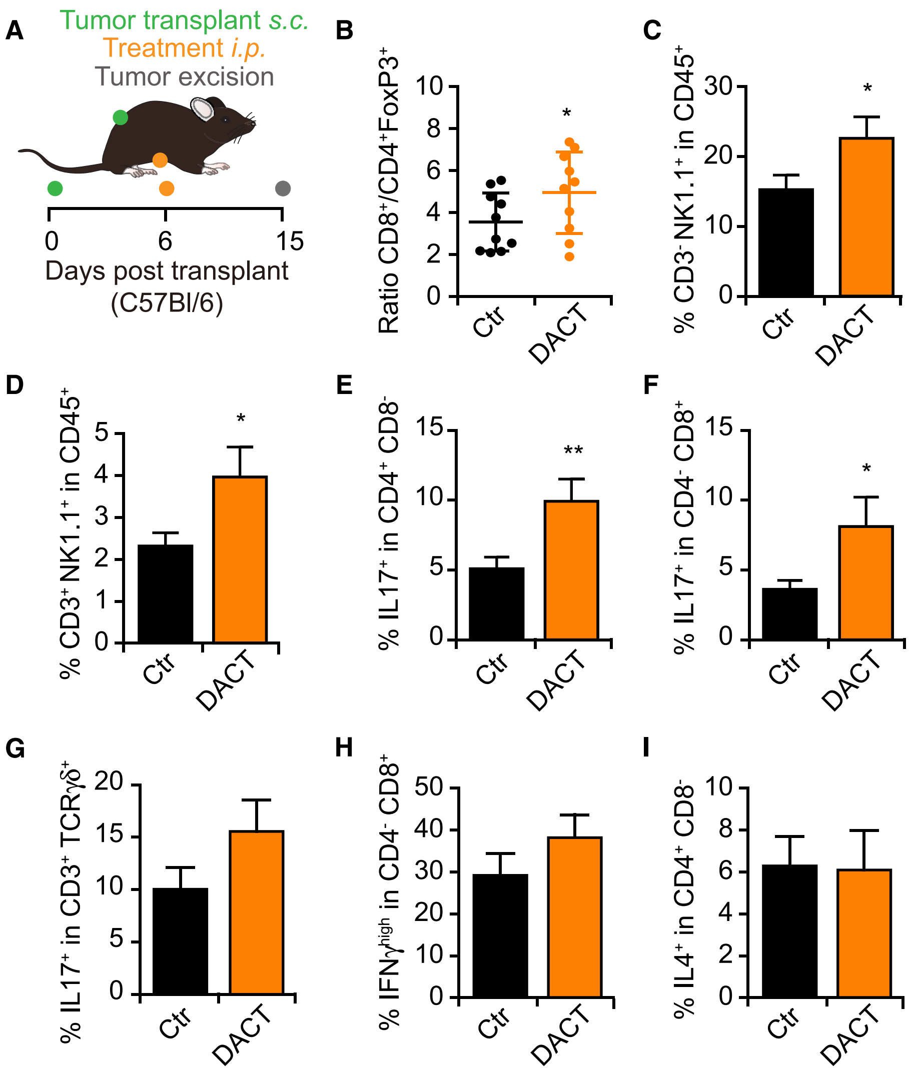

Figure EV3. T-cell immune response induced by dactinomycin.

A–I 2×10^5 mouse fibrosarcoma MCA205 cells were injected subcutaneously (*s.c*) into the flank of immunocompetent syngeneic C57Bl/6 mice (n = 10 per group). When tumors became palpable, injectable solution (Ctr) or 0.5 mg/kg dactinomycin (DACT) was administered intraperitoneally (*i.p.*). Nine days after chemotherapy, the mice were sacrificed, and the tumors were collected and processed (A). 50 mg of tumor was used for each antibody panel, the "*T-cell panel*" with n = 10 mice per group and the "*NK cells and cytokines panel*" with n = 9 mice for Ctr group and n = 8 mice for DACT group. The "*T-cell panel*" included staining of CD4, CD8a, and FoxP3 receptors. A dot plot with means ± SD showing the ratio of the number of $CD3^+CD8a^+$ cells versus the number $CD3^+CD4^+FoxP3^+$ cells in each tumor is depicted with each dot corresponding to one mouse (B). The "*NK cells and cytokines panel*" included CD45, CD3g,d,e, CD8a, CD4, NK1.1, TCRγ(, IL17a, IFNγ, and IL4. The mean ± SEM of the percentage of CD3g,d,$e^-NK1.1^+$ (C) and CD3g,d,$e^+NK1.1^+$ cells (D) among all $CD45^+$ cells is depicted. The mean ± SEM of the percentage of $IL17a^+$-positive cells among the CD3g,d,$e^+CD4^+CD8a^-$ T cells (E), among CD3g,d,$e^+CD4^-CD8a^+$ T cells (F), and among CD3g,d,e^+TCRγ$(^+$ is shown (G). The mean ± SEM of the percentage of $IFN\gamma^{high}$ cells among CD3g,d,$e^+CD4^-CD8a^+$ T cells (H) and of $IL4^+$ cells among CD3g,d,$e^+CD4^+CD8a^-$ T cells (I) is depicted. *P*-values of the statistical difference to the Ctr group were calculated using Student's *t*-test: *$P < 0.05$, **$P < 0.01$ (B–I).

 © 2020 The Authors

Figure EV4. Mechanisms of inhibition of transcription and translation.

A U2OS wild-type, U2OS wild-type treated with 1 μM ISRIB, and U2OS eIF2αS51A cells (three different clones) were used to assess whether the inhibition of translation was dependent on eIF2α phosphorylation. U2OS wild-type and the three U2OS eIF2αS51A clones were pre-treated for 12 h with 3 μM 1 μM dactinomycin (DACT), 1 μM flavopiridol (FLAVO), lurbinectedin (LURBI), 1 μM bortezomib (BTZ), 150 μM cisplatin (CDDP), 10 μM crizotinib (CRIZ), 3 μM daunorubicin (DAUN), doxorubicin (DOXO), 5 μM epirubicin (EPI), 3 μM mitoxantrone (MTX), 500 μM oxaliplatin (OXA), 3 μM paclitaxel (PACL), 3 μM vinblastine (VB), or 3 μM vincristine (VC) or for 12 h (in the presence of ISRIB in the correspondent condition). Cells were further treated with the same drugs or with the controls, 50 μM cycloheximide (CHX) or with 3 μM thapsigargin (THAPS), for 30 min, in methionine-free medium, which was then supplemented with 50 μM L-azidohomoalanine (AHA), a detectable analogue of methionine for an additional 1.5 h before fixation. Images were acquired by fluorescence microscopy, and the percentage of translation inhibition is shown: Fluorescence intensities were ranged between the untreated control (Ctr, 0% inhibition) and a control that was not incubated with AHA (corresponding to 100% inhibition). Results from one representative experiment among three independent ones are shown (mean ± SD of quadruplicates). Statistics were calculated using R pairwise multiple comparisons test with a Benjamin–Hochberg correction: *P*-value comparing each treatment to the control in the wild-type cells is shown with stars; for one treatment, the *P*-values in ISRIB-treated cells and eIF2αS51A clones compared to the wild-type cells are shown with hashes: $^{*/\#}P < 0.05$, $^{**/\#\#}P < 0.01$, $^{***/\#\#\#}P < 0.001$.

B–G We used a Rush (retention using selective hooks) assay consisting of a GFP reporter coupled to a streptavidin-binding peptide (SBP) that in the absence of biotin is retained by a streptavidin expressing hook in the endoplasmic reticulum, in order to evaluate the reversibility of transcription inhibition. Biotin has a high affinity to streptavidin, and its addition leads to the release of the GFP reporter and its secretion via exocytosis. When biotin is removed and leftovers are sequestered by the addition of avidin, the GFP reporter is retained inside the cells (B). Cells were pre-treated with 40 μM biotin for 4 h and with 3 μM doxorubicin (DOXO), 3 μM daunorubicin (DAUN), 5 μM epirubicin (EPI), 3 μM mitoxantrone (MTX), 1 μM bortezomib (BTZ),1 μM dactinomycin (DACT), 10 μM crizotinib (CRIZ), 150 μM cisplatin (CDDP), or 500 μM oxaliplatin (OXA) for 2.5 h. After washout, cells were incubated with 1 μM avidin to assess reversibility of the transcription inhibition (discontinuous treatment, blue line). As a positive control, cells were further treated in the presence of avidin (continuous treatment, green line). Other controls were performed: untreated control (Ctr), control with biotin pre-incubation only (+Biotin), and control with biotin pre-incubation followed by sequestration with avidin (+Avidin) (C). Then, images were acquired every hour for 24 h. Representative images of the different controls, as well as of CRIZ- and DACT (continuous and discontinuous)-treated cells, are shown after background removal at each time point. Scale bar represents 20 μm. (D). Green fluorescence intensity was normalized to biotin-treated controls at each time point, and kinetics is depicted (E). Protein inhibition is represented as slope of the continuous treatment, with the slope of the avidin condition corresponding to 0% inhibition (F). Reversibility is depicted as the area between curves of continuous and discontinuous treatments, with the area between the curve of the control incubated with biotin the curve incubated with biotin and then avidin corresponding to 100% reversibility (G). Barcharts represent means ± SD of three independent experiments upon outlier exclusion. *P*-values evaluating the statistical differences between the inhibition induced by each treatment and the control condition in the presence of avidin (corresponding to 0% inhibition) (F) or between the reversibility of each treatment and the difference between biotin and avidin curves (corresponding to 100% reversibility) (G) were calculated using Student's *t*-test: $^{*}P < 0.05$, $^{**}P < 0.01$, $^{***}P < 0.001$.

© 2020 The Authors

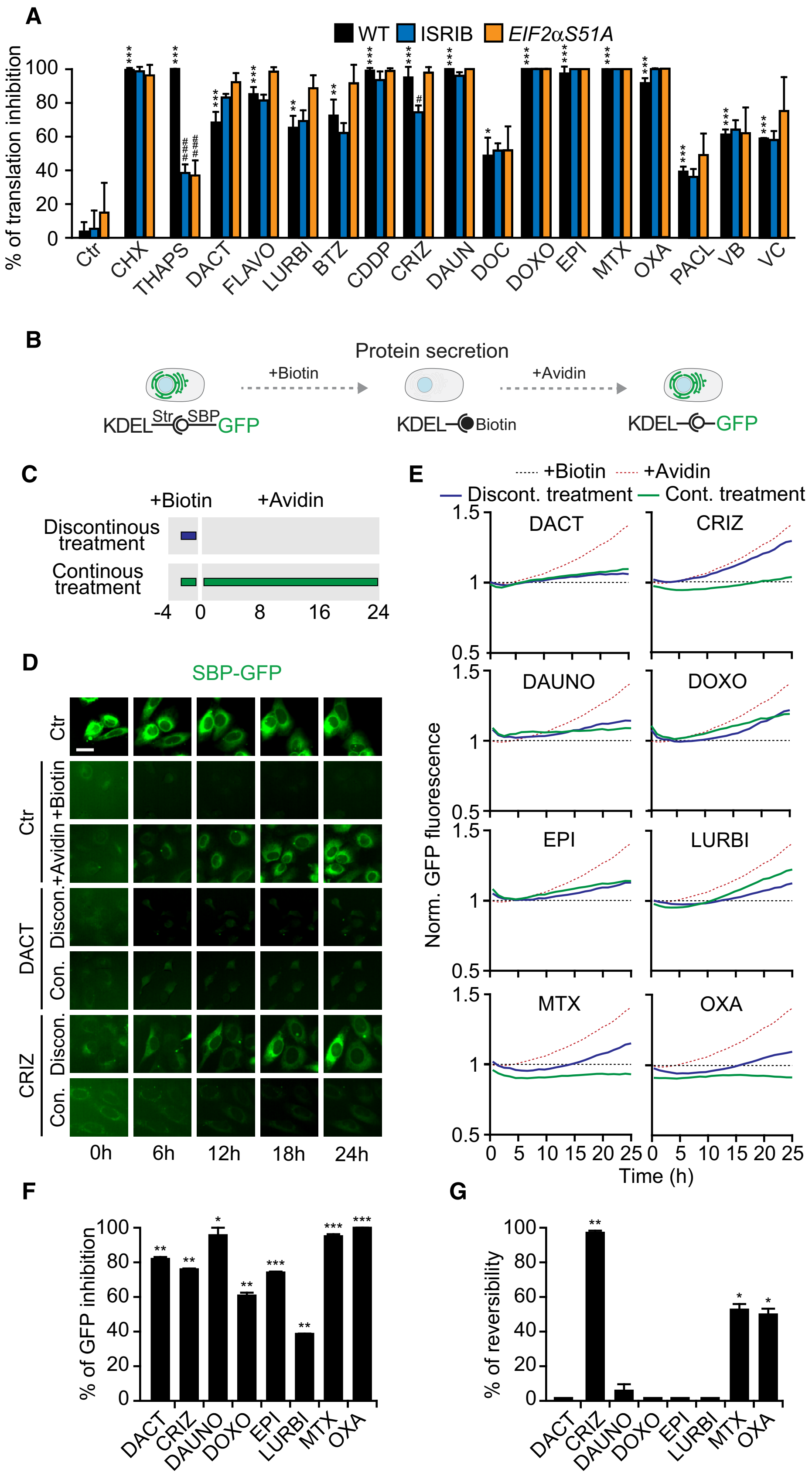

Figure EV4.

 © 2020 The Authors

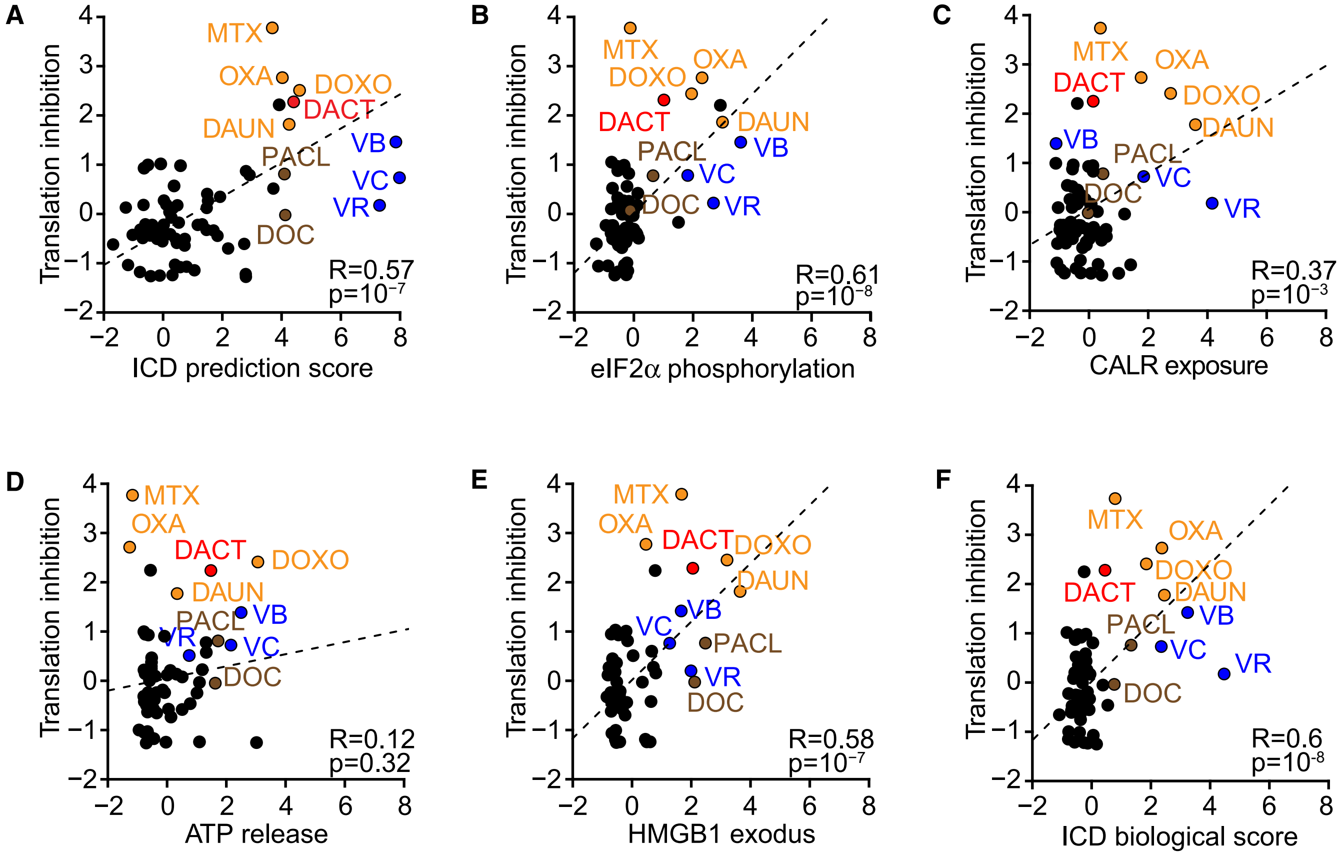

Figure EV5. Translation inhibition correlates with the hallmarks of ICD.

A–F U2OS wild-type cells were pre-treated for 12 h with a custom-made anticancer library as previously described (Bezu *et al*, 2018) at 3 μM followed by 30-min treatment in methionine-free medium, before addition of azidohomoalanine (AHA). The percentages of inhibition of translation were transformed as z-scores. The correlations between translation inhibition and ICD prediction score (A), peIF2α expression (B), CALR exposure (C), ATP decrease (D), HMBG1 decrease (E), and biological calculated ICD score (F) previously measured and also expressed as z-scores (Bezu *et al*, 2018) were compared by means of the Pearson method resulting in a correlation coefficient (R) and corresponding *P*-value (*P*). Known immunogenic drugs are highlighted in color: dactinomycin (DACT), mitoxantrone (MTX), doxorubicin (DOXO), daunorubicin (DAUN), oxaliplatin (OXA), docetaxel (DOC), paclitaxel (PACL), vinblastine (VB), vincristine (VC), and vinorelbine (VR).

© 2020 The Authors
